# Effect of Hot Water Bottles on Body Temperature during Magnetic Resonance Imaging in Dogs under General Anesthesia: A Retrospective Study

**DOI:** 10.3390/vetsci9120660

**Published:** 2022-11-25

**Authors:** Yuki Shimizu, Teppei Kanda, Kenji Kutara, Akihiro Ohnishi, Kaori Saeki, Masahiro Miyabe, Taketoshi Asanuma, Katsumi Ishioka

**Affiliations:** 1 Veterinary Medical Teaching Hospital, Okayama University of Science, Ikoino-oka 1-3, Imabari, Ehime 794-8555, Japan; 2 Graduate School of Veterinary Nursing and Technology, Nippon Veterinary and Life Science University, 1-7-1 Kyonancho, Musashino, Tokyo 180-8602, Japan; 3 Faculty of Veterinary Medicine, Okayama University of Science, Ikoino-oka 1-3, Imabari, Ehime 794-8555, Japan

**Keywords:** body temperature, hypothermia, magnetic resonance imaging, hot water bottle, anesthesia, dog

## Abstract

**Simple Summary:**

In magnetic resonance imaging (MRI) examinations for dogs, general anesthesia or sedation is required for immobilization. It is difficult to prevent hypothermia induced by general anesthesia or sedation due to limitations in body temperature management methods, such as lack of force-warming equipment, including magnetic materials. A hot water bottle (HWB) is one of the few tools that can be brought into an MRI examination room, and can contribute to the prevention or attenuation of hypothermia. In this study, we aimed to retrospectively investigate the effects of HWB on body temperature during MRI examinations in dogs under general anesthesia. We obtained validated data from 100 dogs who underwent MRI under general anesthesia. The decrease in rectal temperature 15 min after intubation was significantly smaller in the group using HWB than in the group not using HWB. Our results suggested that the use of hot water bottles might be one of the methods to attenuate hypothermia in the early period but should not be expected for complete prevention of hypothermia, and it was not recommendable necessarily for body temperature management during MRI examinations in dogs under general anesthesia.

**Abstract:**

Prevention of hypothermia induced by anesthesia and enhanced by low environmental temperatures is difficult in magnetic resonance imaging (MRI) examinations in dogs as forced warming devices, including magnetic materials, are not acceptable for use in the MRI room. A hot water bottle (HWB) can be carried into an MRI examination room and can contribute to the prevention or attenuation of hypothermia. Here, we retrospectively investigated the effects of HWB on body temperature during MRI examinations in dogs under general anesthesia (GA). From anesthesia records of the Veterinary Medical Teaching Hospital, Okayama University of Science, validated data of 100 dogs that underwent an MRI examination under GA were obtained and divided into the following two groups: one group received HWB, while the other did not. Decrease in rectal temperature 15 min after intubation was significantly smaller in the group using HWB than in the group without HWB. In conclusion, the use of hot water bottles might be one of the methods to attenuate hypothermia in the early period but should not be expected for complete prevention of hypothermia, and it was not recommendable necessarily for body temperature management during MRI examinations in dogs under general anesthesia.

## 1. Introduction

In small animal practice, magnetic resonance imaging (MRI) examination requires general anesthesia or sedation in order to ensure that dogs remain motionless during examination, thereby facilitating capture of high-quality images for appropriate diagnosis [1]. However, general anesthesia or sedation causes hypothermia as one of the undesirable effects [2], which can be more serious in MRI examinations than in common surgical operations, the reasons of which are described later. 

Inhalant or injection anesthetics used for MRI examinations cause a physiological decrease in body temperature by suppressing the hypothalamus, which is the center of peripheral vasodilation and thermoregulation [2,3]. Furthermore, physical heat losses due to radiation, conduction, convection, and evaporation enhance the decrease in body temperature under general anesthesia or sedation [4]. As hypothermia can cause a variety of adverse events, including decreased drug metabolism, organ dysfunction, and abnormalities in the immune system, it is necessary to prevent hypothermia or to reduce the decrease in body temperature during MRI examination under general anesthesia or sedation [3]. 

There are two major reasons for the increased severity of hypothermia or decrease in body temperature during MRI examinations. First, in recent veterinary practice, various methods have been adopted to prevent hypothermia during anesthetic treatment, such as use of a forced-air warming system, a heat-retaining mat, and intravenous infusion warming devices, in addition to classical methods such as hot water bottles or blankets [5,6]. These devices are effective and safe to use in a place without restrictions, such as a surgical theater. However, these devices cannot be used in an MRI examination room due to the inclusion of magnetic materials. Therefore, methods to prevent hypothermia during MRI examinations are limited. The second reason is that the room temperature of the MRI examination room is kept low to maintain the equipment [7]. Low-temperature environments enhance hypothermia due to convention; cold air surrounds the body surface causing heat loss [3,4,8]. Thus, during MRI examinations, a lower room temperature worsens hypothermia induced by general anesthesia.

Therefore, prevention or attenuation of hypothermia in anesthetized dogs during MRI examination is recognized as a challenging issue. In veterinary medicine, only Onozawa et al. have reported the effect of an insulation device in preventing hypothermia during MRI examinations in dogs and cats under general anesthesia [9]. In human pediatric medicine, similar to veterinary medicine, hypothermia during MRI examination is regarded as a serious problem because children who have difficulty remaining still during an MRI scan may require sedation or general anesthesia to minimize movement. Previous reports have shown that hypothermia is common in anesthetized children undergoing MRI: methods to prevent hypothermia have not been addressed [10,11]. We were unable to obtain useful information from human medicine. Based on scientific evidence, there are no standard or recommended methods to prevent or attenuate hypothermia in anesthetized dogs during MRI examinations, although hot water bottles, blankets, or other insulations are customarily used.

The use of hot water bottles is a possible way to prevent or attenuate anesthesia-induced hypothermia in an MRI examination room. Recently, although it is a method not often used in the operating room, a hot water bottle was used to mitigate hypothermia during anesthesia and to promote recovery from hypothermia [12,13]. A hot water bottle that does not contain magnetic material, such as a resin bottle or rubber glove filled with hot water, can be safely brought into an MRI room. In Onozawa’s study, hot water bottles were used to prevent hypothermia in dogs and cats during MRI examinations. This study reported the insulative effect of bubble wrap and down blankets covering hot water bottles in dogs and cats during MRI examinations, although the effect of hot water bottles themselves on body temperature was not investigated [9]. However, there have been no reports on the effect of hot water bottles during MRI examinations on body temperature of animals or their role in prevention or alleviation of hypothermia.

Therefore, in the present study, we aimed to retrospectively investigate the effect of hot water bottles on body temperature during MRI examinations in dogs under general anesthesia.

## 2. Materials and Methods

This was a retrospective, observational study. The subjects of this study were included from the anesthesia records of dogs that underwent MRI examinations at the Veterinary Medical Teaching Hospital, Okayama University of Science (OUS-VMTH), from November 2018 to May 2021.

In this study, dogs meeting the following inclusion criteria were included: (1) those that underwent MRI examinations under general anesthesia; (2) those with a pre-anesthetic rectal temperature (Pre-RT) between 37 and 40 °C; (3) those with the American Society of Anesthesiologists Physical Status (ASA-PS) classification of 1 to 3; (4) those that underwent MRI examinations for more than 60 min; and (5) those for which hot water bottles and a shredded paper comforter, or only a shredded paper comforter was applied for body temperature management. The anesthetic records of dogs that met these inclusion criteria were used as data for retrospective observations. Dogs were excluded from the study even if one of the inclusion criteria was not met.

The hot water bottle in this study was an infusion solution bag containing 500 mL of water, which was heated in a 600 W microwave oven for 90 s before use. The cases in which hot water bottles were applied met two conditions: (1) the two hot water bottles were wrapped in separate face towels—to avoid the risk of burn injuries—and placed on the left and right sides of the dog’s abdomen; and (2) the hot water bottle was not replaced throughout the MRI examination duration. The paper comforter used in the study was paper shredded by a shredder packed in a plastic bag. Cases in which the paper comforter was applied met the following condition: a paper comforter was placed over the dog, covering from the neck to the tip of the tail.

The following information was extracted from the target data: breed, ASA-PS, sex, age, body weight (BW), Body Condition Score (BCS), rectal temperature (RT) at each time point, sedatives or anesthetics administered, and dose.

To analyze the effect of the hot water bottles on body temperature, cases were divided into the following two groups: the hot water bottle (HWB) group, including dogs treated with both, a hot water bottle and a paper comforter for body temperature management, and the shredded paper comforter (SPC) group, including dogs treated with a SPC alone. For further analysis, dogs from the HWB and SPC groups were assigned to subgroups based on their BW and RT before induction of anesthesia (Pre-RT). Dogs with Pre-RT < 38.5 °C or >38.5 °C were designated as HWB-L or SPC-L and HWB-H or SPC-H groups, respectively. Dogs with BW < 5 or >5 kg were designated as HWB < 5 or SPC < 5 and HWB ≥ 5 or SPC ≥ 5 groups, respectively.

The variables sex, age, BCS, ASA-PS, and RT were considered for statistical analyses, performed using GraphPad Prism (9.0, GraphPad Software, Inc., San Diego, CA, USA). Sex was compared between the groups or subgroups using Fisher’s exact test. The continuous variables, RT at each measurement point, age, and weight, and categorical variables, BCS and ASA-PS, were compared between the HWB and SPC groups using the Mann–Whitney test. RT at each measurement point, age, weight, BCS, and ASA-PS were compared between subgroups using Kruskal–Wallis analysis of variance on ranks and Dunn’s multiple comparison test. The Friedman and Dunn’s multiple comparisons test were used to compare RT at each measurement point and Pre-RT within each group or subgroup. Statistical significance was set at *p* < 0.05.

## 3. Results

### 3.1. Demographics

The total number of animals used in this study was 100. The cases included 57 males and 43 females, aged 115 (3–201) months [median (range)], weighing 5.6 (0.7–40) kg, with an evaluated BCS of 5 (2–8), and ASA of 2 (1–3). The dogs belonged to the following 23 breeds (number of dogs): Mongrel dog (16), Miniature Dachshund (16), Chihuahua (14), Toy Poodle (13), French Bulldog (7), Beagle dog (4), Shih Tzu (3), Pembroke Welsh Corgi (3), Pug (3), Miniature Schnauzer (3), Golden Retriever (2), Shiba Inu (2), Boston Terrier (2), Pomeranian (2), Yorkshire Terrier (2), Tea Cup Poodle (1), English Cocker Spaniel (1), Kaninchhen Dachshund (1), Jack Russell Terrier (1), Chinese Crested Dog (1), Papillon (1), Border Collie (1), and Miniature Pinscher (1).

Before anesthesia induction, each dog was premedicated with butorphanol (butorphanol 5 mg, Meiji Seika Pharma Co., Ltd., Tokyo, Japan), midazolam (Dorumicum Injection 10 mg, Maruishi Co., Ltd., Osaka, Japan), both, or none. General anesthesia was induced with propofol (1% 20 mL intravenous propofol injection “Pfizer,” Maruishi Co., Ltd., Osaka, Japan) or Alfaxalone (Alfaxan, Meiji Seika Pharma Co., Ltd., Tokyo, Japan), and the dog’s trachea was intubated (Table 1). After intubation, general anesthesia was maintained with sevoflurane delivered in oxygen via a rebreathing system.

As per the above-mentioned criteria, 50 dogs were assigned to each HWB and SPC group and divided into subgroups (Table 2). There were no statistically significant differences in sex, age, BCS, or ASA-PS between the HWB and SPC groups; however, BW showed a significant difference. No statistical differences in sex, age, BCS, ASA-PS, or weight were found between the HWB-L, HWB-H, SPC-L, and SPC-H subgroups and the HWB < 5, HWB ≥ 5, SPC < 5, and SPC ≥ 5 subgroups. There were significant differences in body weight between the HWB < 5 and HWB ≥ 5, HWB < 5 and SPC ≥ 5, SPC < 5 and HWB ≥ 5, and SPC < 5 and SPC ≥ 5 subgroups.

All MRI examinations were performed with a 1.5 T superconducting machine (Vantage Elan, Canon Medical Systems, Otawara, Japan), and the MRI room temperature was maintained at 23 ± 1 °C throughout the investigation period. Image artifacts caused by the hot water bottles were not reported.

### 3.2. Rectal Temperature

There were significant differences in Pre-RT between the HWB-L and HWB-H groups, the SPC-L and SPC-H groups, the HWB-L and SPC-H groups, and the HWB-H and SPC-L groups (Table 3). In contrast, no statistical differences in Pre-RT were found between the HWB and SPC groups, HWB-L, SPC-L, HWB-H, and SPC-H subgroups, and HWB < 5, SPC < 5, HWB ≥ 5, and SPC ≥ 5 subgroups.

RT decreased significantly from T15 to T60 in the HWB and SPC groups compared to Pre-RT. There were no statistically significant differences in RT between the HWB and SPC groups at any time point. However, when the difference between Pre-RT and RT calculated at each time point (ΔT15-, 30-, 45-, and 60-Pre), ΔT15-Pre in the HWB group (−0.4 ± 0.3 °C) was significantly smaller than that in the SPC group (−0.6 ± 0.5 °C).

In the HWB-H group, RT at T15 did not significantly decrease compared to Pre-RT. In the HWB-L, SPC-L, and SPC-H groups, RT decreased significantly from T15 to T60 compared with Pre-RT. In addition, in the HWB-H subgroup, RT significantly decreased from T30 to T60 compared with Pre-RT.

In the HWB-H and HWB-L subgroups at T15, RT was higher than that in the SPC-H and SPC-L subgroups, respectively. In addition, at T15, RTs were higher in the HWB-H group than in the HWB-L group and in the SPC-H subgroup than in the SPC-L subgroup. At T30, RT was higher in HWB-H than in HWB-L, in SPC-H than in SPC-L, in HWB-H than in SPC-L, and in SPC-H than in HWB-L. At T60, there were no statistically significant differences in RT among the HWB-L, HWB-H, SPC-L, and SPC-H subgroups.

In the HWB < 5, HWB ≥ 5, SPC < 5, and SPC ≥ 5 groups, RT decreased significantly from T15 to T60 compared to Pre-RT. There were no statistically significant differences in the RT between the HWB < 5, SPC < 5, HWB ≥ 5, and SPC ≥ 5 subgroups at any time point.

### 3.3. Safety

No adverse events associated with the hot water bottle placement—including burn injuries—occurred during the MRI examinations.

## 4. Discussion

In both the HWB and SPC groups, the RT decreased significantly within 30 min after anesthesia induction, regardless of body temperature or BW before induction. After anesthesia induction, the decrease in body temperature occurs in three phases: the redistributable (phase 1), linear (phase 2), and plateau (phase 3) phase [14]. In phase 1, administration of anesthetics caused redistribution of heat due to peripheral vasodilation, resulting in a rapid decrease in core temperature within 1 h after the onset of general anesthesia. Thus, our results suggest that hot water bottles could not prevent a decrease in RT induced by redistribution of heat with anesthetic administration, such as propofol or alfaxalone, for MRI examinations [15,16].

However, the decline in RT at 15 min after anesthetic induction in the HWB group was significantly smaller than that in the SPC group. Furthermore, in the HWB-H subgroup, RT did not decrease significantly for 15 min after anesthetic induction, although the SPC-H subgroup showed a significant decrease at the same time point. The RT at 15 min after intubation was significantly higher in the HWB-H and HWB-L than in the SPC-H and SPC-L subgroups, respectively. There were no statistically significant differences in body temperature before anesthesia between the HWB-H and SPC-H groups and the HWB-L and SPC-L groups. These results suggest that hot water bottles attenuated the decrease in RT for 15 min after intubation. At the same time, it was also suggested that the effect of hot water bottles was sustained for about 15 min. Frequent renewal of hot water bottles may be required to sustain their effect on body temperature. However, in clinical practice, frequent bottle renewals are limited.

Between the SPC-L and SPC-H subgroups, a significant difference in RT was found 15 min after intubation. It has been suggested that RT before anesthesia also affects the maintenance of body temperature. In human medicine, pre-warming aimed at increasing the body temperature before anesthesia induction was reported to reduce the incidence of hypothermia during general anesthesia; even if hypothermia is not completely prevented, hypothermia may be mitigated [17,18,19]. However, a study on dogs reported that pre-warming was not useful for maintaining body temperature during general anesthesia and remained controversial [20,21].

The RT and BCS did not significantly differ between the HWB/SPC < 5 and HWB/SPC ≥ 5 subgroups. These results suggest that BW did not affect the body temperature attenuation by hot water bottles in the absence of differences in BCS. In general, the smaller the animal, the larger the surface area-to-body mass ration, thereby promoting greater heat loss by convection [22,23]. Namely, dogs in the HWB/SPC < 5 subgroups lost their heat more easily than dogs in the HWB/SPC ≥ 5 subgroups. Thus, we expected the RT in the HWB/SPC < 5 subgroups to decrease more than that in the HWB/SPC ≥ 5 subgroups; however, such was not the case.

In the present study, administered drugs, including sedatives, anesthetics, and supportive drugs, were not considered in the analysis. Thus, the influence of different drugs on the effect of hot water bottles on body temperature in dogs was not discussed. This is a limitation of the present study.

## 5. Conclusions

Our retrospective investigation suggested that the use of hot water bottles might be effective for body temperature management in dogs during MRI examinations under general anesthesia, especially in the early period after anesthesia induction, although hypothermia could not be prevented. However, the attenuating effect of the hot water bottles on the decrease in body temperature was minimal and the risk of burn injuries could not be completely ruled out. The use of hot water bottles might be one of the methods to attenuate hypothermia in the early period but should not be expected for complete prevention of hypothermia, and it was not recommendable necessarily for body temperature management during MRI examinations in dogs under general anesthesia.

## Figures and Tables

**Table 1:** Anesthetic and sedative drugs administered for anesthetic management.

Drugs	Number of Dogs	Dose (mg/kg)
Premedication		
midazolam	7	0.2 (0.2–1.9)
butorphanol	41	0.2 (0.1–1.4)
midazolam + butorphanol	6	0.2 (0.2–1.7) + 0.2 (0.2–1.4)
none	46	-
Induction		
Alfaxalone	73	2.2 (0.4–6.1)
propofol	27	4.0 (0.9–7.3)

The number of dogs and dose of drugs are indicated as the real number and median (range), respectively.

**Table 2:** Demographic data of each analyzed group and subgroup.

	HWB				SPC			
	HWB-L	HWB-H	HWB < 5	HWB ≥ 5	SPC-L	SPC-H	SPC < 5	SPC ≥ 5
Number of dogs	50				50			
	33	17	26	24	21	29	18	32
Age (months)	104 ± 51				113 ± 44			
	105 ± 51	101 ± 52	95 ± 53	116 ± 49	121 ± 40	108 ± 46	113 ± 48	114 ± 42
Sex (male/female)	31/19				26/24			
	19/14	10/7	14/12	15/9	13/8	13/16	10/8	16/16
BW (kg)	5.7 ± 3.7 ^b^				8.1 ± 6.5 ^a^			
	5.7 ± 3.8	5.4 ± 3.2	3.2 ± 1.2 ^i,j^	8.5 ± 3.4 ^g,h^	7.3 ± 3.6	8.7 ± 8.0	3.5 ± 1.0 ^i,j^	10.6 ± 7.0 ^g,h^
BCS	4 (2–7)				4 (2–8)			
	4 (3–7)	4 (2–7)	4 (2–7)	5 (4–7)	4 (2–6)	5 (3–8)	4 (2–6)	5 (3–8)
ASA–PS	2 (1–3)				2 (1–3)			
	2 (1–3)	2 (1–3)	2 (1–3)	2 (1–3)	2 (1–3)	2 (1–3)	2 (1–3)	2 (1–3)
Pre-RT (°C)	38.3 ± 0.4				38.4 ± 0.4			
	38.0 ± 0.2 ^e,f^	38.8 ± 0.3 ^c,d^	38.3 ± 0.4	38.3 ± 0.4	38.0 ± 0.2 ^e,f^	38.7 ± 0.2 ^c,d^	38.4 ± 0.3	38.5 ± 0.4

Number of dogs and sex (male/female) were indicated as real numbers, BCS and ASA-PS were indicated as median (range), and age (months), BW (kg), and Pre-RT (°C) were indicated as mean ± SD.. Each superscript character means significant differences at each time point compared to the (^a^) HWB and (^b^) SPC groups, and the (^c^) HWB-L, (^d^) HWB-H, (^e^) SPC-L, (^f^) SPC-H, (^g^) HWB < 5, (^h^) SPC < 5, (^i^) HWB ≥ 5, (^j^) SPC ≥ 5 subgroups (*p* < 0.05).

**Table 3:** Rectal temperature in each group and subgroup.

	Time after Intubation			
Treatments	Pre	T15	T30	T60
HWB	38.3 ± 0.4	37.9 ± 0.5 *	37.2 ± 0.6 *	37.0 ± 0.8 *
HWB-L	38.0 ± 0.2 ^e,f^	37.5 ± 0.3 *^,d,e^	37.0 ± 0.6 *^,e,f^	36.8 ± 0.8 *
HWB-H	38.8 ± 0.3 ^c,d^	38.3 ± 0.4 ^c,d,f^	37.6 ± 0.5 *^,c,d^	37.4 ± 0.6 *
HWB < 5	38.3 ± 0.4	37.8 ± 0.5 *	37.2 ± 0.7 *	37.0 ± 0.7 *
HWB ≥ 5	38.3 ± 0.4	37.8 ± 0.6 *	37.2 ± 0.6 *	36.9 ± 0.8 *
SPC	38.4 ± 0.4	37.7 ± 0.6 *	37.2 ± 0.6 *	37.1 ± 0.7 *
SPC-L	38.0 ± 0.2 ^e,f^	37.4 ± 0.5 *^,c,e,f^	36.9 ± 0.5 *^,e,f^	36.8 ± 0.5 *
SPC-H	38.7 ± 0.2 ^c,d^	37.9 ± 0.6 *^,d,f^	37.4 ± 0.6 *^,c,d^	37.3 ± 0.7 *
SPC < 5	38.4 ± 0.3	37.6 ± 0.6 *	37.0 ± 0.6 *	36.8 ± 0.7 *
SPC ≥ 5	38.5 ± 0.4	37.7 ± 0.6 *	37.4 ± 0.5 *	37.2 ± 0.5 *

* means of significant differences in RT compared to Pre-RT within each group or subgroup (*p* < 0.05). Each superscript character means significant differences at each time point compared to the (^c^) HWB-L, (^d^) SPC-L, (^e^) HWB-H, and the (^f^) SPC-H subgroups (*p* < 0.05).

## Data Availability

Not applicable.
